# Supplementary material for: Analysis of PPARγ Signaling Activity in Psoriasis
Source: Int J Mol Sci. 2021 Aug 10;22(16):8603. doi: 10.3390/ijms22168603 (PMC8395241; doi:10.3390/ijms22168603)
Supplement: Supplementary file 1 [file ijms-22-08603-s001.zip › Supplemental materials_Analysis of PPARg signaling activity in psoriasis/Pathway models/Models images and html files/Interleukin-17 and interleukin-22 signaling in psoriasis/1000616342.html]

PIP3 --+> PDPK1


# DirectRegulation PIP3 --+> PDPK1

|  |  |
| --- | --- |
| URN | urn:agi-directregulation:in-out:urn:agi-cas:165689-82-7:out:urn:agi-llid:5170:eff=positive:mch=direct%20interaction |
| Connectivity | 2 |
| References | 558 |
| Effect | positive |
| Mechanism | direct interaction |
| ObjectType | DirectRegulation |
| RelationArity | 2 |
| RelationNumberOfReferences | 239 |
| RelationSymbolicName | PIP3}OID --+> PDPK1}OID |
| RelationConfidenceLevel | 3 |
| RelationParticipantTypes | Small Molecule -> Protein |
| Original # of References | 157 |

---
